# Swedish local and regional politicians’ views on their role in health promotion

**DOI:** 10.1093/eurpub/ckac130.222

**Published:** 2022-10-25

**Authors:** S Svanholm, H Carlerby, E Viitasara

**Affiliations:** Department of Health Sciences, Mid Sweden University, Sundsvall, Sweden

## Abstract

**Background:**

The political context is an important determinant of health. Politicians in municipalities and regions in Sweden are responsible for many of the determinants of health, and their role is therefore important when considering health promotion. The aim of the study was to explore how politicians describe their role in health promotion.

**Methods:**

An electronic questionnaire focused on politicians’ role, responsibility, and possibility to promote health was sent to all politicians in municipalities and regions in the north of Sweden. A total of 667 politicians answered the questionnaire, and out of them, 361 politicians answered the free text question “as a politician I consider my role in health promotion to be ...”. The answers were analyzed using thematic analysis. The four themes discovered were used to sort politicians into groups. All politicians were sorted into the group that was most similar to their answer. Group sizes were shown in percentage of how large part of the politicians belonged to the respective groups.

**Results:**

Preliminary results show that the politicians could be divided into four different groups: 1) No political role, only personal aspects described (such as being a good role model) (25,3%), 2) Promote individuals to take care of their health (for example through information) (19,5%), 3) Support other parts of the organization (municipality or region) to promote health, mainly through financial support and agenda-setting (29,8%), 4) Most (if not all) political decision-making affect health (25,4%).

**Conclusions:**

There is a large variety in how politicians describe their role in promoting health. Only approximately half of the politicians see that their political decision-making can directly affect the health of the population. With the political context being an important determinant of health, this could be considered a missed opportunity for structural health promotion work.

**Key messages:**

- Approximately half of the politicians did not describe political means of affecting population health.
- Not recognizing the political determinants of health risk making politicians miss the opportunity for structural health promotion work.

